# A Tympanal Insect Ear Exploits a Critical Oscillator for Active Amplification and Tuning

**DOI:** 10.1016/j.cub.2013.08.028

**Published:** 2013-10-07

**Authors:** Natasha Mhatre, Daniel Robert

**Affiliations:** 1 School of Biological Sciences, University of Bristol, Bristol BS8 1UG, UK

## Abstract

A dominant theme of acoustic communication is the partitioning of acoustic space into exclusive, species-specific niches to enable efficient information transfer. In insects, acoustic niche partitioning is achieved through auditory frequency filtering, brought about by the mechanical properties of their ears [1]. The tuning of the antennal ears of mosquitoes [2] and flies [3], however, arises from active amplification, a process similar to that at work in the mammalian cochlea [4]. Yet, the presence of active amplification in the other type of insect ears—tympanal ears—has remained uncertain [5]. Here we demonstrate the presence of active amplification and adaptive tuning in the tympanal ear of a phylogenetically basal insect, a tree cricket. We also show that the tree cricket exploits critical oscillator-like mechanics, enabling high auditory sensitivity and tuning to conspecific songs. These findings imply that sophisticated auditory mechanisms may have appeared even earlier in the evolution of hearing and acoustic communication than currently appreciated. Our findings also raise the possibility that frequency discrimination and directional hearing in tympanal systems may rely on physiological nonlinearities, in addition to mechanical properties, effectively lifting some of the physical constraints placed on insects by their small size [6] and prompting an extensive reexamination of invertebrate audition.

## Highlights

• The tympanal ears of a tree cricket use active amplification
• Active amplification and not passive resonance determines tuning to song frequency
• Active amplification and tuning have an “on” and an “off” state
• Crickets are the phylogenetically oldest insects with active auditory amplification

## Results and Discussion

Sensory cells in vertebrate ears expend energy to enhance auditory acuity by actively amplifying their mechanical response to sound [7–12]. Some insect ears, the antennae of fruit flies and mosquitoes, also possess such active amplification, a physiological mechanism analogous to that of mammalian tympanal ears [2, 13, 14]. Antennal ears are unconventional sound receivers [10]; they respond to particle velocity rather than sound pressure, exhibit lower mechanical inertia than do tympanal ears, and are populated with a large number of mechanosensory cells, from 500 in *Drosophila* [15] to 16,000 in mosquitoes [16, 17], many more than in tympanal ears [18] (but see [19]). While the sensory and motile ciliated cells of *Drosophila* can produce sufficient power to drive ear mechanics [12], it is uncertain whether cells in other chordotonal organs produce forces and whether those are sufficiently powerful to impart motion to the tympanal structure [5]. To date, the question of active amplification in tympanal insects remains unresolved [5]. The suggestion that tympanal ears exhibit active amplification is based solely on the observation of acoustically recorded distortion products during two-tone stimulation [20]. Distortion products, however, may be produced even in the absence of active amplification [21] and on their own do not constitute unambiguous proof of active amplification. Additionally, acoustic observations of nonlinear amplification from both locust and moth auditory systems could not be confirmed using the more sensitive technique of laser Doppler vibrometry (LDV) under free-field acoustic stimulation [5, 22].

In this study, we reveal the existence of the canonical, well-defined hallmarks characteristic of actively amplified systems [10, 23, 24], using noncontact Doppler vibrometry under free-field quantitatively controlled acoustic conditions. We show that active auditory amplification is present in the tympanal auditory organ of a tree cricket, an orthopteran insect.

### The Tree Cricket Tympanum Oscillates Spontaneously

**Figure 1:**
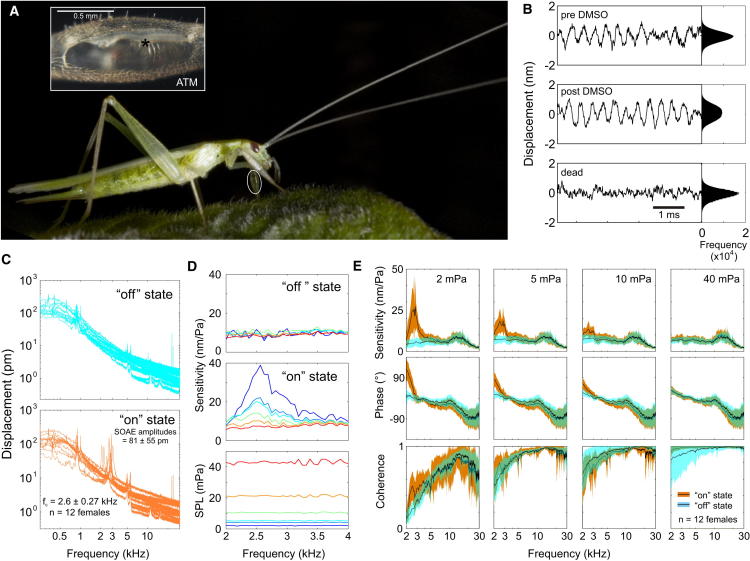
The Auditory System of *Oecanthus henryi*, Spontaneous Oscillations and the “On” and “Off” Response states (A) The position of the auditory organ is marked on the foreleg of a female tree cricket. The organ includes two membranes, the anterior (ATM) and posterior (PTM) tympanal membranes (see Figure S1 for further details). The larger ATM is depicted in the inset, with an asterisk marking the position of maximum measurement coherence. (B) The ATM oscillates spontaneously even in the absence of sound stimulation. A frequency distribution of ATM displacements over 25 s exhibits a unimodal pattern, in contrast to the bimodal pattern observed in hair cells and insect flagellar ears. The distribution broadens upon administration of DMSO. No periodic oscillations are observed upon death, revealing a narrower distribution. (C) Spectral composition of spontaneous oscillations (SOs). Variations in SO frequency and amplitudes are observed across individuals (n = 12 animals, mean and ± 1 SD shown; f_c_ range 2.1 to 3.0 kHz; amplitude range 11.3 to 193.8 pm; Table S1). Notably, SO f_c_ is restricted to the frequency range of conspecific song. Interestingly, SOs are not constitutively switched on (“on” state) but emerge from previously quiescent ATMs (“off” state) (see also Figure S2 and Table S1). (D and E) The mechanical sensitivities of the same ATMs were measured using periodic chirps, in both the “on” and “off” state. In the “off” state, the ATM showed no evidence of tuning in the frequency range of conspecific song and its sensitivity was linear (D) for a single individual (color code applies to all three subfigures, with cooler colors indicating lower SPLs and warmer colors higher SPLs) and (E) across a population (dark line shows the mean, shaded zone indicates 1 SD). In the “on” state, the same ATM exhibits tuning at low SPLs. This tuning is compressively nonlinear and SPL dependent; the peak in sensitivity becomes progressively smaller and broader as stimulus SPL increases, both in (D) individual responses and (E) across a population (see also Figure S3). (E) Compressive nonlinearity is observed only within the conspecific frequency range and at higher frequencies sensitivity is always independent of stimulus amplitude. See also Figures S1–S3 and Table S1.

Each foreleg of a tree cricket is endowed with two tympanal membranes, the anterior and the posterior. Both show similar mechanical behavior [25]. While measuring the mechanical response of the larger anterior tympanal membrane (ATM) in the tree cricket *Oecanthus henryi* (Figure 1A and Figure S1 available online), we discovered that it oscillated spontaneously, even in the absence of sound stimulation (Figures 1B and 1C). The oscillations did not show the clear bimodal frequency distributions observed in other systems [26] even after the administration of DMSO (Figure 1B); however, they did disappear upon death (Figure 1B). These oscillations were restricted to a small frequency band, which lay within the range of the conspecific song (Figure 1C; 2.63 ± 0.27 kHz, mean ± SD, range 2.08 to 3.02 kHz; song 2.3 to 3.7 kHz [27]). Interestingly, spontaneous oscillations in the conspecific frequency range could be measured from previously quiescent ATMs (Figure 1C). These oscillations, though small (81.1 ± 55.6 pm, mean ± SD, n = 12), were well above the noise floor measured prior to their spontaneous appearance. The noise floor decreased from 12.21 ± 3.98 pm at 2 kHz to 7.29 ± 2.18 pm at 3 kHz (mean ± SD, n = 12); further details on “on” and “off” states are given in the Supplemental Information (Table S1 and Figure S2). The oscillations were transiently suppressed only by tones at nearby frequencies supporting their active origin (Figure S2). Additionally, their amplitudes were not reduced by the administration of glutamate, suggesting that force generation was independent of muscle function (Figure S2). The emergence of spontaneous oscillations in a biologically relevant frequency range supports the notion that the oscillations are driven by a physiological mechanism, such as the motility of mechanosensory cells.

In effect, these oscillations may be a form of spontaneous otoacoustic emissions (SOAEs) produced by a critical oscillator-like mechanism analogous to those observed from vertebrate hair cells [28] as well as the antennal ears of mosquitoes [2] and fruit flies [3, 29]. Among the theoretical frameworks used to explain auditory mechanics are the so-called “critical oscillators” (COs), which explain the emergence of SOAEs from the activity of mechanosensory cells [11, 23, 30, 31]. In insects, the specific implementation of an auditory CO appears to be a generalized van der Pol oscillator [23, 31]. The action of this oscillator manifests itself as a force that reduces damping in a displacement-dependent manner [30, 31]. When a harmonic force is applied to the oscillator, deflection disproportionate to the applied force is seen, but only at the extremes of displacement, i.e., at phase 90° and −90°. The feedback force that causes this excess displacement, can maintain the oscillator at the threshold of an oscillatory instability, i.e., at its “critical” point, such as a Hopf bifurcation point [23]. Remarkably, under the governing physics, this critical operating point can be perturbed by mechanical forces just above thermal noise. Such thermal perturbations can set the oscillator into spontaneous oscillation, but only at a specific frequency, known as its critical frequency (f_c_) [10, 23].

We hypothesize that the SOAEs observed from the tree cricket ATM are a manifestation of such a CO-based instability. To substantiate this argument, we tested for the presence of a series of features diagnostic of an actively amplified CO-based auditory system: compressive nonlinearity, two-tone suppression accompanied by distortion products, and, finally, physiological vulnerability [10, 23, 24].

### The Tympanum Shows Compressive Nonlinearity

Conventionally, a system with CO-based active amplification displays the sharpest frequency tuning and highest gain at the lowest stimulus levels [23], endowing ears with high sensitivity and frequency selectivity. As stimulus level increases, both sharpness and peak sensitivity decrease, effectively increasing the system’s dynamic range, a phenomenon termed compressive nonlinearity [23]. To test for this behavior, we presented multifrequency stimuli at different sound pressure levels (SPLs) and measured the ATM response with LDV. Measurements were carried out on the same individuals both when no SOAEs were observed and when they emerged subsequently (Figures 1D and 1E). In all 12 individuals tested, the tympanal response was linear in the absence of SOAEs (“off” state; Figures 1D and 1E). The frequency response of the same ATM became compressively nonlinear upon the emergence of SOAEs (“on” state; Figures 1D and 1E). In the “on” state, at low SPLs, the ATM was clearly tuned and a peak in sensitivity was observed within the range of song frequencies (Figures 1D and 1E). As SPL was increased, mechanical sensitivity to song frequencies decreased and tuning became less sharp, until it reached the previously reported broad sensitivity [25, 32] (Figures 1D and 1E).

Compressive nonlinearity was confined only to a small frequency range below 4 kHz (Figures 1D and 1E). The low-frequency response could be fitted to a simple harmonic oscillator model with an f_0_ of 2.73 ± 0.15 kHz (mean ± SD at 2 mPa stimulation, R^2^ = 0.85 ± 0.06, n = 12 females, “on” state; Figure S3). The observed passive membrane resonance, however, occurred at a much higher frequency f_0_ = 16.02 ± 1.56 kHz (mean ± SD, R^2^ = 0.78, n = 12 females, “off” state; Figure S3) and showed no compressive nonlinearity. The ATM sensitivity in the “off” state resembled the high SPL response in the “on” state, supporting the idea that it reflected the membrane’s passive resonance (Figure 1E). In fact, in the “off” state, ATM sensitivity did not display any tuning to conspecific song frequency or compressive nonlinearity (Figures 1D and 1E). However, both clear compressive nonlinearity and spontaneous oscillations could be observed later in the day from the same tympanal membrane of the same individuals (Figures 1D and 1E). Hence, these data showed that active amplification and not passive resonance determined tuning to conspecific song frequency (Figure 1E). Remarkably, nonlinear mechanical sensitivity and tuning could be switched “on” or “off” (Figures 1D and 1E).

### The Change in Gain Follows the Power Law Predicted for Critical Oscillators

**Figure 2:**
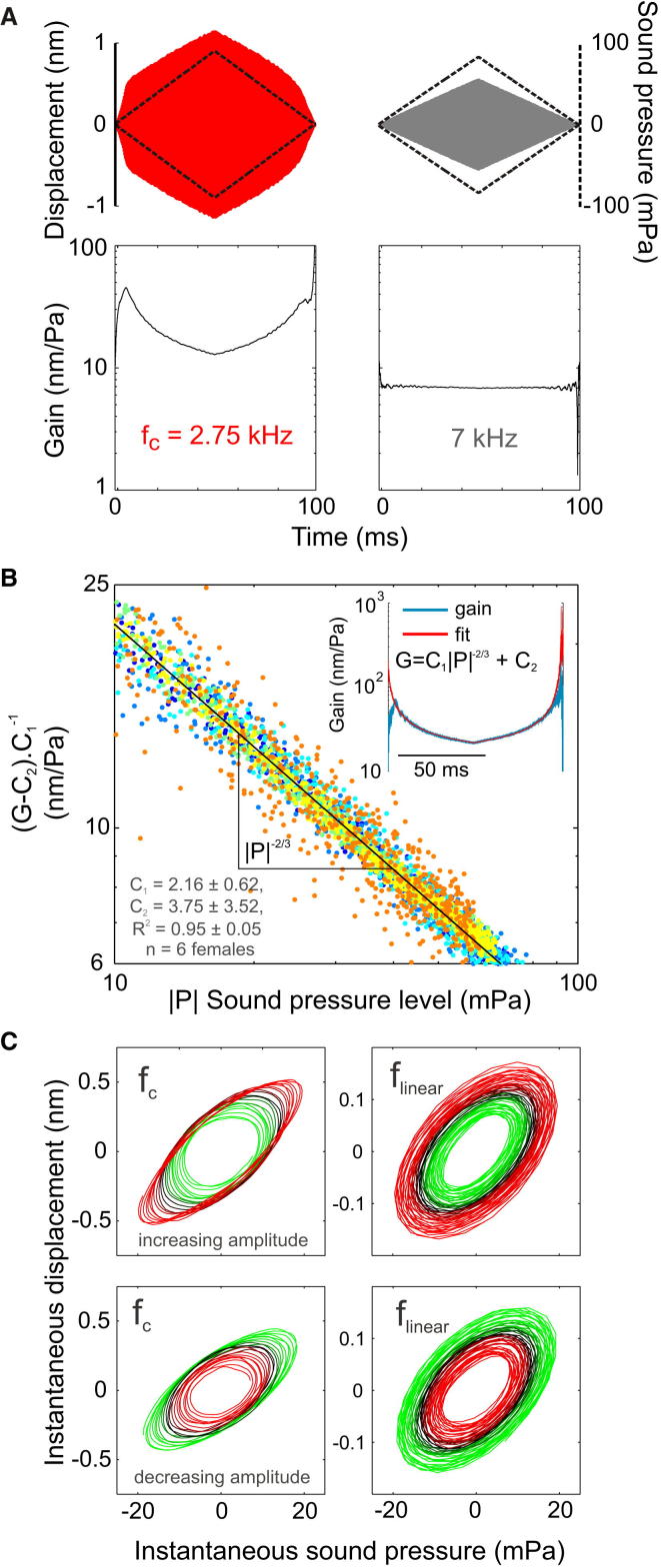
Level and Phase-Dependent Gain at f_c_ (A) ATM displacement over time (solid-colored envelopes) in response to amplitude modulated single tone stimuli (black stippled line envelopes) demonstrates that gain is level dependent near f_c_ but constant at 7 kHz. (B) Near f_c_, gain varies with SPL following the power law dependence (G = C_1_|P|^−2/3^) predicted for COs when a term for the linear passive oscillator is included (C_2_). Each color indicates a different individual. The inset shows gain from a single female fitted to the modified equation (additional data in Figure S4). (C) Lissajous plots display instantaneous displacement of the ATM against the instantaneous sound pressure (colored to facilitate visualization of different trajectories). At 7 kHz, the Lissajous plot is concentric and the distances between trajectories are equal at all radial axes indicating a fixed proportionality between sound pressure and displacement. Near f_c_, however, the separation between the trajectories varies and is greatest at ± 90° displacement phase. This demonstrates that the active force that increases ATM displacement is phase dependent as in a van der Pol oscillator. See also Figure S4.

The change in CO sensitivity near a Hopf bifurcation can be described in mathematical terms. At f_c_, the gain (|x| / |f|) of the CO decreases with stimulus amplitude [23] by

(1) $$\frac{\left|\text{x}\right|}{\left|\text{f}\right|}∼{\left|\text{f}\right|}^{-2\text{/}3}\text{,}$$

where x is the displacement amplitude and f is the magnitude of the acting force. In order to test whether the tree cricket ATM response fits this equation, we recorded ATM response to an amplitude modulated stimulus at f_c_. It was then compared to the response at 7 kHz, a frequency that lies in the linear regime. We found that the gain was amplitude dependent (Figure 2A). Gain changed as predicted by Equation 1 at f_c_, when additional linear terms accounting for the passive ATM response were included (Figure 2B). This finding supports the conjecture that the amplitude dependent oscillator was a CO near a Hopf bifurcation and was in interaction with a linear oscillator (the ATM). Slight hysteresis was observed in the gain, and we therefore also tested the SPL dependence of the ATM gain using 100 ms pure tone pulses, which showed similar results (Figure S4).

We also analyzed the ATM response using a Lissajous plot, displaying the instantaneous sound pressure against instantaneous displacement (Figure 2C), hence revealing the phase of the force applied by the active mechanism. Lissajous plots were generated for low SPLs, in the regime where gain was highest. ATM displacement was plotted in response to single frequency sound stimulation between 10 and 20 mPa, with both increasing and decreasing amplitude, at f_c_ and at 7 kHz (Figure 2C).

In the linear case, at 7 kHz, displacement amplitude increased proportionally to sound amplitude (Figure 2C). The resulting Lissajous plot was concentric and the distances between trajectories were equal at all radial axes (Figure 2C). At f_c_, however, the separation between plot lines varied and was greatest at the displacement phase +90° and −90° (Figure 2C), a diagnostic feature of van der Pol oscillator behavior [33]. The observed excess displacement suggested that the ATM response at this phase was not driven by the sound pressure alone, and the applied active forces were made apparent. This finding was supported by the observation that damping ratio increased with SPL, as predicted for a van der Pol oscillator (Figure S3). Thus, tree cricket tympanal ears show some notable similarities to both *Drosophila* [31] and mosquito auditory systems [34], suggesting some shared underlying mechanisms. However, some important differences are apparent. For instance, in contrast to *Drosophila* [35], in tree crickets, f_0_, which corresponds to f_c_, does not vary much with SPL (Figure S3).

### Two-Tone Stimulation Near f_c_ Produces Two-Tone Suppression and Distortion Products

When stimulated by two tones simultaneously, COs oscillate at a series of additional frequencies, the so-called distortion product otoacoustic emissions (DPOAEs). The amplitudes of these distortion products decay exponentially [23, 36]. In addition to distortion products, two-tone suppression can also be observed. Two-tone suppression is the decrease of gain at f_c_ that occurs when f_c_ is presented along with another tone of similar frequency. The presence of distortion products can however be artifactual; it has been shown that if a system is driven at high amplitudes, distortion products can be produced even where no compressive nonlinearity is present [21]. Hence, the production of DPOAEs only in the presence of low stimulus levels in conjunction with compressive nonlinearity satisfies predictions of CO-like behavior [23, 36].

**Figure 3:**
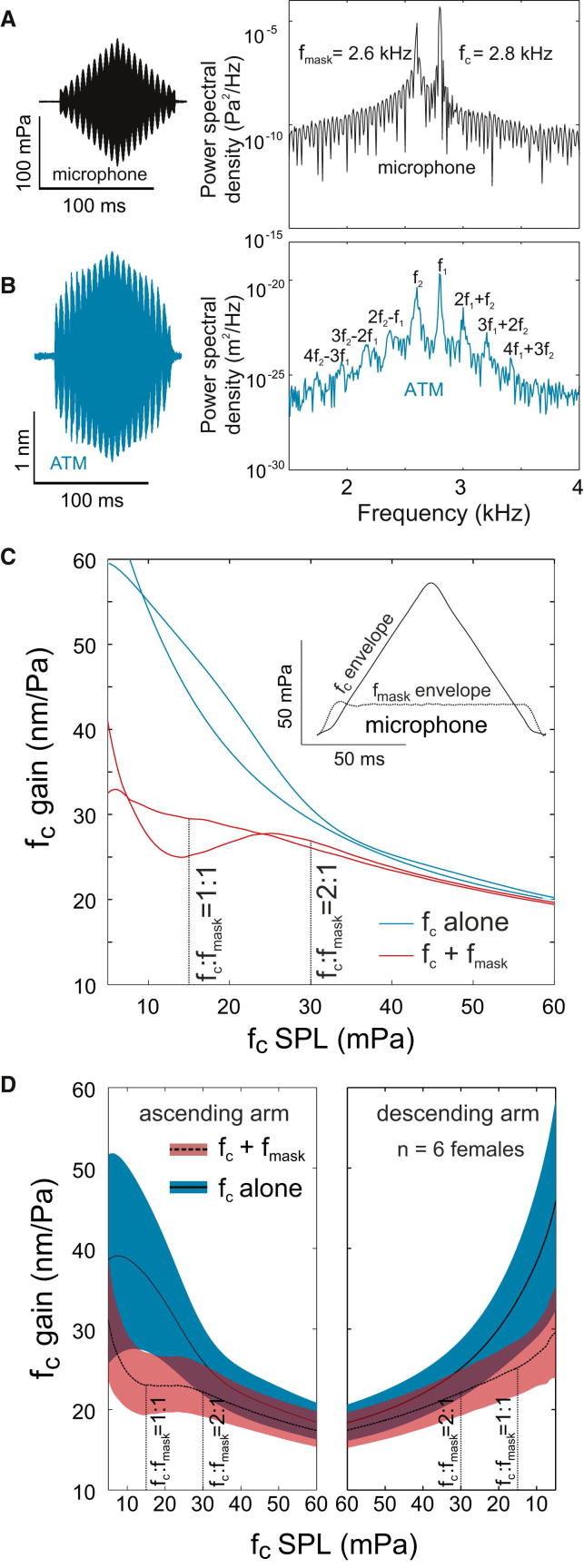
DPOAEs and Two-Tone Suppression Time and frequency representations of (A) the two-tone analytical stimulus (note absence of distortion products in sound) and (B) the ATM mechanical response exhibiting DPOAEs. The gain of the ATM response at f_c_ is lower in the presence of a masking tone than when presented alone. This difference is greatest when the ratio of f_c_: f_mask_ is less than 2 and at higher ratios, the two gains begin to converge. This behavior, called two-tone suppression, can be observed both (C) in individual measurements and (D) across a population (mean ± SD is shown). The inset shows the relative levels of the critical (f_c_) and masking frequency (f_mask_ = f_c_ − 200 Hz) in the acoustic stimulus. Since hysteresis is observed, the gain resulting from ascending and descending amplitude ramps are displayed separately. See also Figure S4.

Hence, we tested for DPOAEs only near f_c_ using low-amplitude stimuli. We also measured whether distortion products are accompanied by two-tone suppression, allowing us to test for the behavior of a true CO-like nonlinear amplifier [21, 23, 36]. ATM displacement was recorded in response to analytical signals that contained a main tone near f_c_ and a masking tone 200 Hz above or below the main tone (Figures 3A and S4). As expected, exponentially decaying DPOAEs were observed in the ATM response, even when absent in the sound stimulus (Figures 3A, 3B, and S4). In order to allow us to simultaneously test for two-tone suppression, we designed the analytical signal so that the level of the main tone varied continuously with respect to the fixed level masking tone. The response to each tone could be assessed by filtering both sound stimulus and ATM responses for that tone and calculating gain. Gain suppression at the main tone was observed when the ratio of the main to masking tone levels was between 0:1 and 1:2 (Figures 3C and 3D). Like SPL-dependent gain, both DPOAEs and two-tone suppression were also observed using 100 ms constant amplitude stimuli (Figure S4), further substantiating the presence of CO based active amplification in the tree cricket ear.

### Physiological Vulnerability of Active Amplification

Many oscillatory systems can exhibit nonlinear behavior [33, 37]. In some cases, this behavior is caused by driving a system with excessive power and placing it beyond its elastic limits, into a region of nonlinear, non-Hookean stress-strain relationship [37]. In other cases, the applied force need not be excessive; it is the very structure of the oscillator that causes nonlinear behavior—for instance, piecewise oscillators in which the oscillator makes intermittent contact with components are inherently nonlinear [37]. However, based on the similarity between the tree cricket and other actively amplified auditory systems and the presence of active amplification in conspecific song frequency range, we propose that the present nonlinearity is an adaptation for enhancing signal detection and that it emerges from a physiological mechanism. In order to substantiate this claim, we demonstrate its physiological vulnerability.

**Figure 4:**
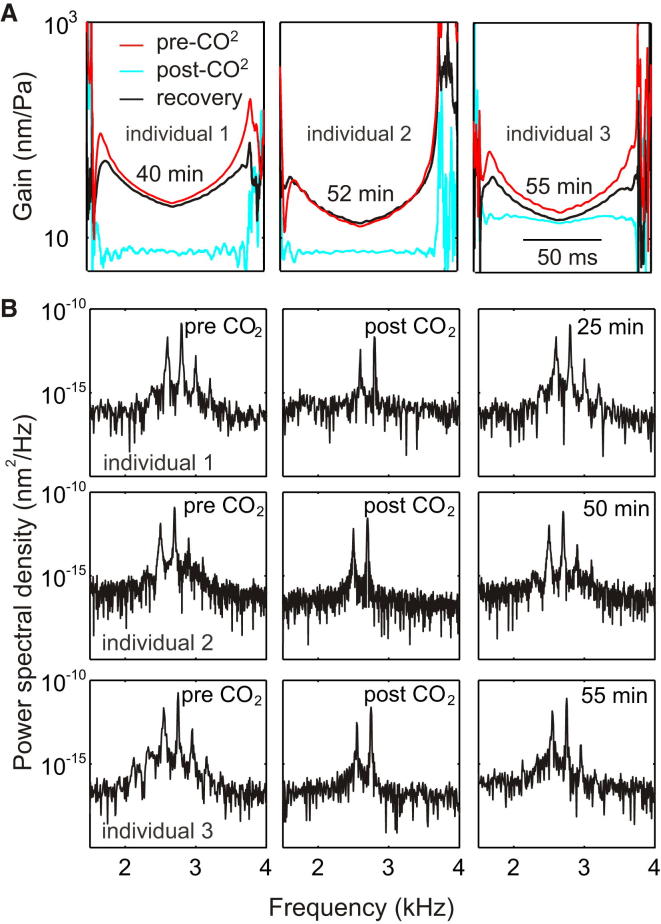
The Effects of Anoxia on Active Mechanics (A) Prolonged hypoxia induced by CO_2_ exposure causes reversible suppression of nonlinear amplification at f_c_ (n = 3). (B) DPOAEs are observed pre-CO_2_ exposure and after recovery, but not immediately after (n = 3). Recovery times are displayed in the figures and vary across individuals. See also Figure S2.

CO_2_-induced hypoxia is known to affect active amplification in both mosquito and *Drosophila* auditory systems [2, 13, 29]. Unlike in mosquitoes and flies, however, hypoxia-induced changes in tree cricket hearing require extended exposure. Exposure for less than 30 min did not reliably abolish nonlinear responses, perhaps attributable to both the poikilothermic nature and larger size of the animal which imply longer diffusion times [38]. When exposed to CO_2_ over 30 min, however, both nonlinear gain at f_c_ (Figure 4A) and DPOAEs (Figure 4B) were abolished. These changes were reversible, fully recovering over time (Figures 4A and 4B). A physiological basis was additionally supported by data from dead tympana (Figure S2). Altogether, the evidence supports a physiological basis for the CO-like nonlinear amplification observed in tree cricket ears, rather than a non-Hookean mechanical nonlinearity.

Tree crickets (Oecanthines) belong to the order Orthoptera, the oldest terrestrial organisms known to possess auditory capabilities [18, 39]. The present experimental evidence makes it apparent that the tree cricket’s tympanal ears can be driven by CO-like active amplification. Functionally, active amplification is required for tuning to conspecific frequencies, while the passive response is clearly not tuned to the conspecific song. The sharpness of mechanical auditory tuning at low SPL raises the question of sender-receiver tuning mismatch in the face of temperature driven changes in male song [27, 32]. Surprisingly, auditory amplification in these insects is flexible. For the first time, evidence emerges for an “on/off” switch for active auditory amplification, serving tuning to conspecific song. Active amplification can now be regarded as a phylogenetically basal trait with previously underappreciated functionality and flexibility.

## Author Contributions

N.M. carried out all experiments and analysis. Both authors contributed to the writing of the manuscript.

## Supplemental Information

Document S1. Supplemental Experimental Procedures, Figures S1–S4, and Table S1
