# Supplementary material for: A Tympanal Insect Ear Exploits a Critical Oscillator for Active Amplification and Tuning
Source: Curr Biol. 2013 Oct 7;23(19):1952–7. doi: 10.1016/j.cub.2013.08.028 (PMC3793861; doi:10.1016/j.cub.2013.08.028)
Supplement: Document S1. Supplemental Experimental Procedures, Figures S1–S4, and Table S1 [file mmc1.pdf]

Current Biology, Volume *23*

Supplemental Information

A Tympanal Insect Ear

Exploits a Critical Oscillator

for Active Amplification and Tuning

Natasha Mhatre and Daniel Robert

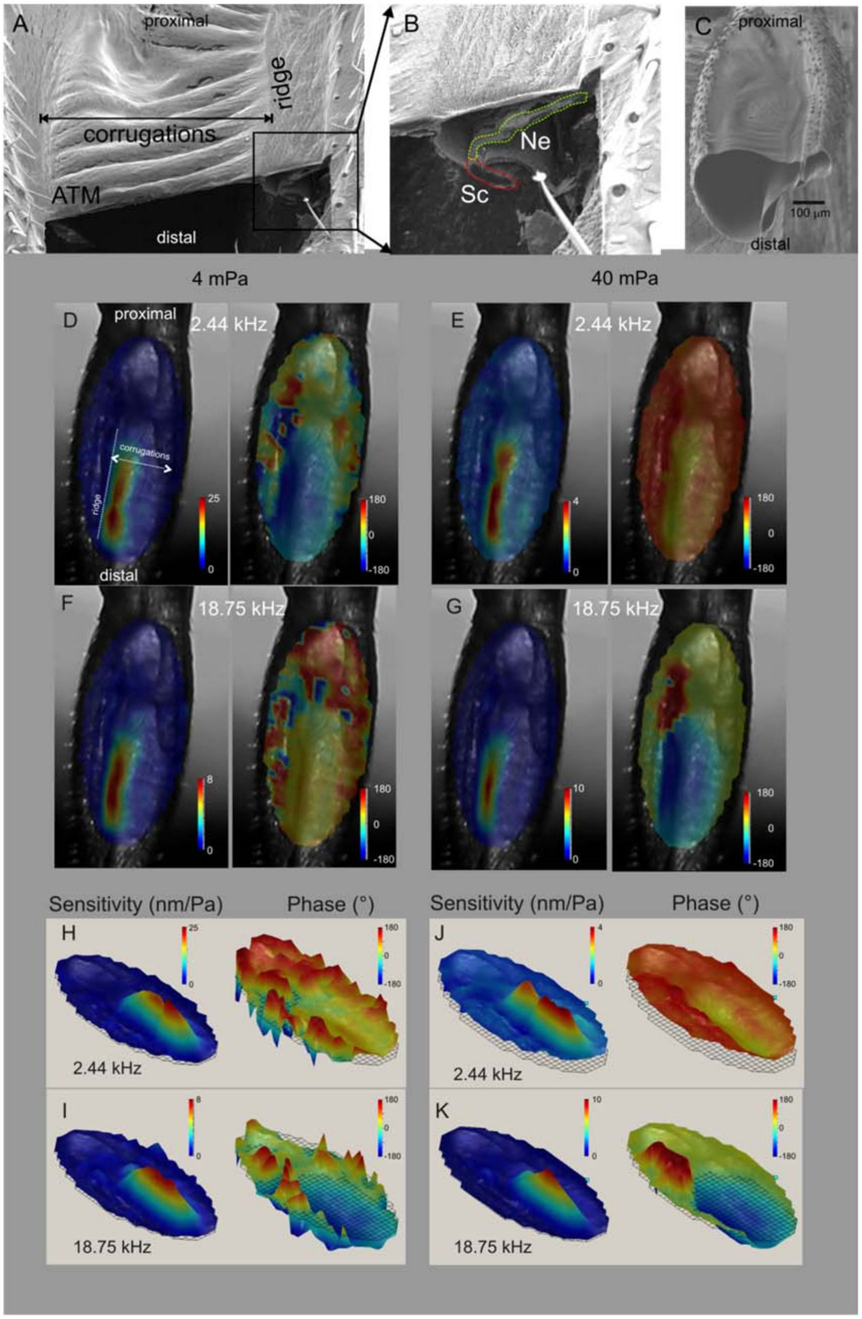

Figure S1. Related to Figure 1. Tree cricket ear anatomy and its relationship to vibration patterns in 2D and 3D (A) A cut through the ATM (**Fig. 1**) using a dual beam focussed ion beam milling station (FEI Helios Nanolab 600) revealing (B) an auditory sensillum within the tracheal organ (Sc: scolopidial capsule (red outline), Ne: bipolar ciliated neuron(green outline)). The position of the sensory cells in tree crickets is analogous to that in other gryllids. Scolopidia are not directly attached to the tympanal membranes. (C) Section of the tree cricket leg near the hearing organ showing that the scolopidia are attached to the tracheal walls, which are in turn attached to the tympanal membranes. Mechanically, a distension of the tracheal walls would cause a distension of the tympanal membranes, allowing the cells to indirectly produce oscillations of the membrane.

In response to sound, the displacement of the ATM is concentrated on the distal half of the ridge where ATM corrugations converge near the scolopidia attachment. Both spontaneous oscillations and coherent displacements are observed from the same position on the ATM. This is true whether the ATM is responding to (D, E, H, J) low or (F, G, I, K) high frequencies or (D, F, H, I) low or (E, G, J, K) high amplitudes. However, the high frequency response has an anti-node around which the displacement shows a phase transition suggesting this is the 2nd mode of the membrane.

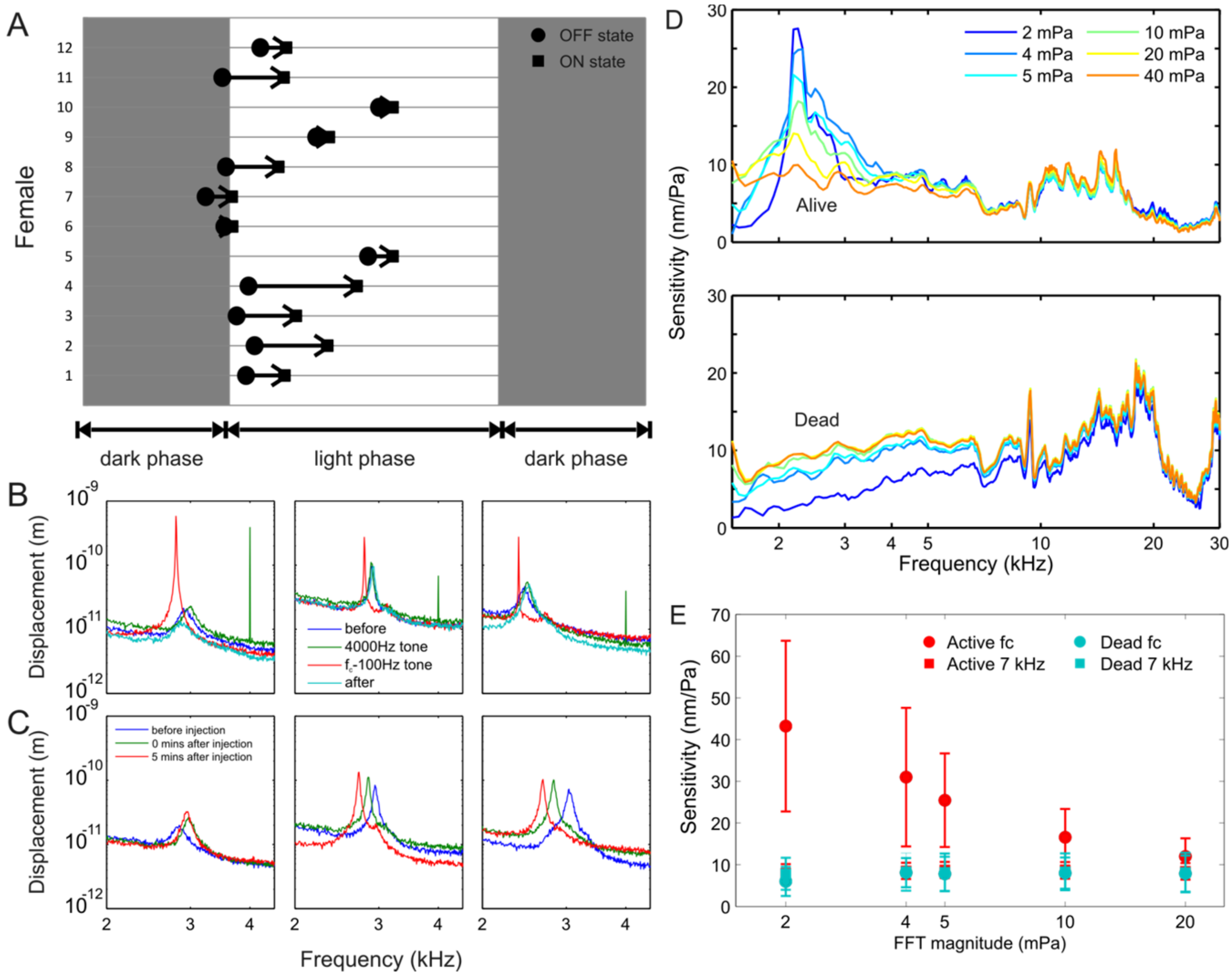

Figure S2. Related to Figures 1 and 4. Physiological basis of active amplification (A)Timings of measurement of OFF and ON state in ATM active mechanics with respect to female light:dark phase (**Fig. 1**). Each horizontal trace marks the time at which the two states were measured for each female. The circle indicates the observation of the OFF state and the square marks the first observation of the ON state. In each case the OFF state was observed before the ON state indicating that the OFF state was not caused simply by death or physiological breakdown. We have found that the OFF state occurs mainly in the light phase of the animals light:dark cycle hinting at the possibility of a circadian rhythm. However, in all tested animals, active amplification returns to the ON state before the onset of the dark phase which marks the onset of their normal activity period. This suggests that the switch is not purely circadian but can be influenced by external environmental stimuli such as sound, light or temperature or even the stress of being handled and mounted.

(B) The observed spontaneous emissions were transiently suppressed when a tone of frequency 100 Hz lower than the SOAE frequency was presented, however were unaffected by tones at higher frequencies (n=3). (C) Similarly, an injection of glutamate, which is known to suppress muscle function, did not reduce the amplitude of the SOAEs, suggesting that muscles were not the force generating mechanism (n=3).

(D) Clear compressive non-linearity around conspecific song frequency can be seen in the same female when alive but not when dead supporting a physiological basis (**Fig. 4**). The ATM frequency response when dead resembles the response in ‘OFF’ state with only a high frequency component (Fig. 1). (E) This loss of compressive non-linearity at f_c can be observed across a population of females (n=5).

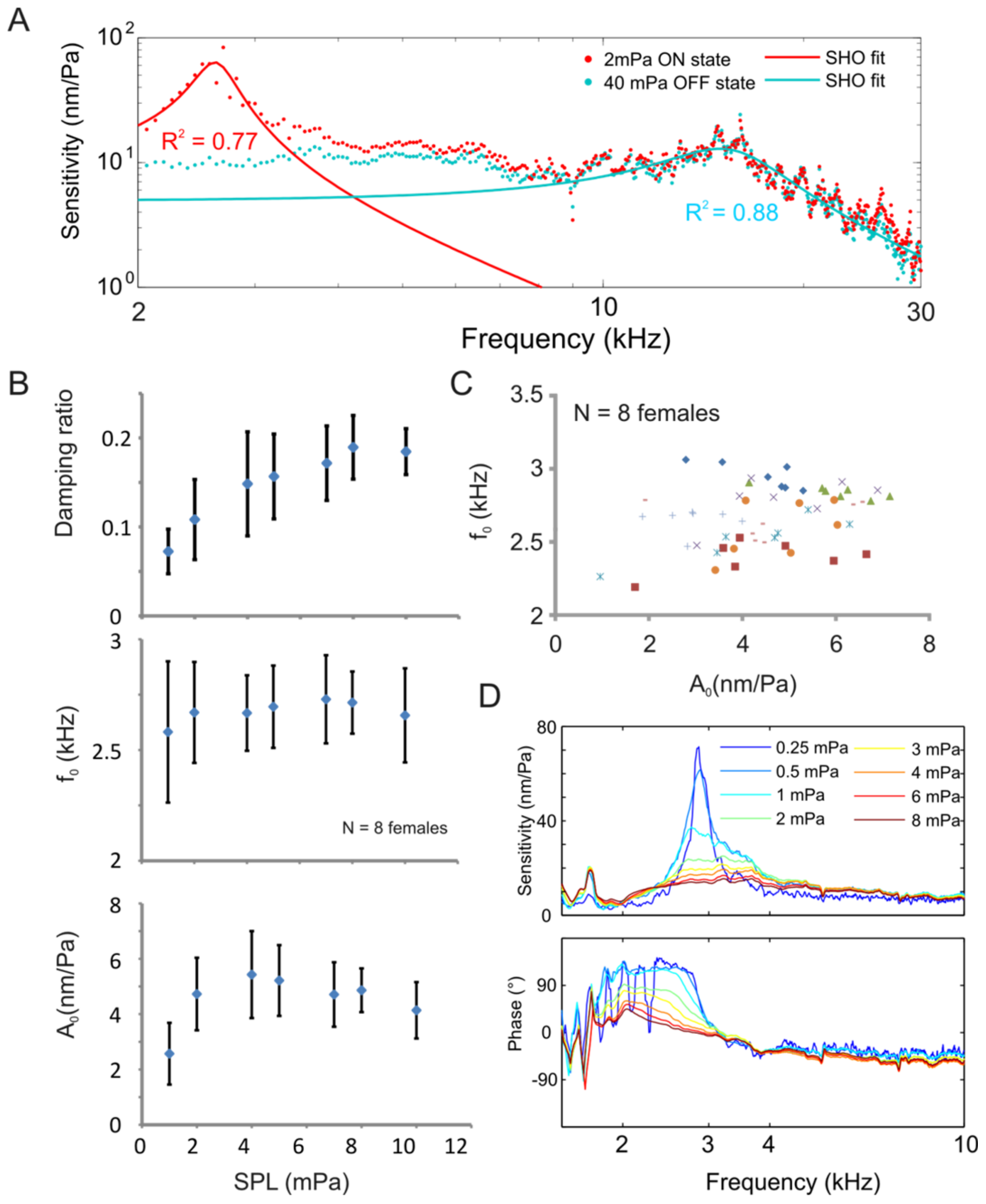

Figure S3. Related to Figure 1. Critical oscillator behaviour (A) Simple harmonic oscillator model (SHO) fits to ATM frequency response. The Active low frequency response in the ON state at 2mPa from a single individual (red) can be fitted to an SHO model. So can the passive high frequency response at 40 mPa in the OFF state from the same individual (blue). Notably, the high frequency response of the tympanal membrane remains unchanged between ON and OFF states (**Fig. 1**).

(B) Fits to the tympanal responses of a set of 8 females measured at a greater number of SPL intervals show that the damping ratio increases with SPL, however, the critical frequency does not appear to change greatly. (C) It is expected that A_0 will decrease when f_0 increases since A_0= F/k and $f_0 = \sqrt{k/m}$, where k is stiffness, F is the applied force and m is mass. However, there is no dependence between A_0 and f_0 and the observed variation is a result of noise within the measurement chain (each symbol represents one individual). (D) Measurements at higher frequency resolution (12.5 Hz) where no change in f_0 is observed as expected for a van der Pol oscillator, as suggested by time domain behaviour (**Fig. 2**).

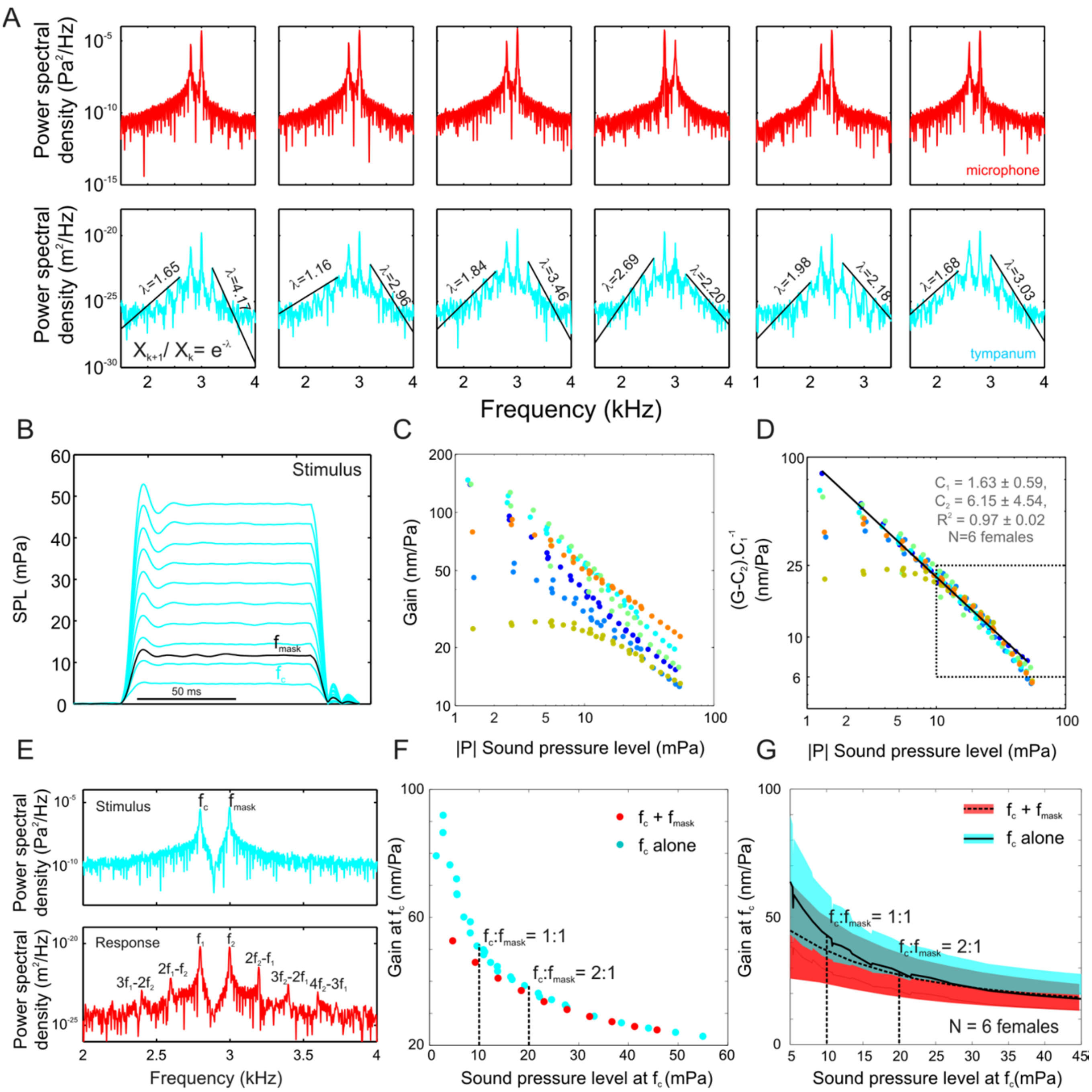

Figure S4. Related to Figures 2 and 3. Two-tone suppression and distortion products (A) DPOAEs recorded from the tympanal vibrations of 6 individuals using the amplitude modulated ramp protocol (**Fig. 3**). The black lines indicate the exponents that describe the decay of the DPOAE amplitudes[1].

(B) The constant level 100 ms stimulus, at f_c and f_{mask}, used to reconfirm the presence of two tone suppression and DPOAEs as seen in the ramped stimuli (**Fig. 3**). Level is calculated as the average from stimulus onset to offset. Response gain is calculated in a similar manner from ATM displacement. (C) Gain at f_c from 6 individuals, each colour represents a single female. In some but not all measurements the ATM gain becomes linear below 5 mPa SPL. (D) As with data from the amplitude modulated stimuli, gain varies with SPL following the power law dependence ($G=C_1|P|^{-2/3}$) predicted for COs when a term (C2) for the linear passive oscillator is included (Complete equation: $G=C_1|P|^{-2/3} + C_2$).

(E) When f_c is presented in conjunction with a masking tone ($f_1 = f_c$, $f_2 = f_{mask}$), the ATM response contains a series of DPOAEs as observed with the amplitude modulated stimuli. A reduction in gain at f_c is also observed when both tones are presented together in (F) individual responses, as well as (G) across a population (Shaded regions indicate one standard deviation around the mean indicated by the data lines).

Individual	SO Frequency (kHz)	SO amplitudes (pm)	Time (OFF state) (hrs)	Time (ON state) (hrs)	Time elapsed (hrs)	Temperature (OFF state) (°C)	Temperature (ON state) (°C)	Temperature difference (°C)
**1**	3.02	91.78	11:41	13:15	01:34	22.90	23.10	0.20
**2**	2.65	15.35	12:02	15:00	02:58	23.13	23.59	0.46
**3**	2.82	72.57	11:18	13:43	02:25	22.98	23.57	0.59
**4**	2.78	53.29	11:47	16:12	04:25	21.48	22.60	1.12
**5**	2.87	193.75	16:40	17:40	01:00	23.07	23.02	-0.05
**6**	2.66	121.91	10:48	11:07	00:19	20.30	20.50	0.20
**7**	2.84	34.43	10:02	11:06	01:04	20.20	21.30	1.10
**8**	2.47	44.62	10:52	13:00	02:08	21.25	21.34	0.09
**9**	2.08	92.70	14:33	15:04	00:31	21.55	21.64	0.09
**10**	2.23	84.11	17:07	17:40	00:33	21.94	22.05	0.11
**11**	2.66	157.13	10:43	13:13	02:30	21.74	21.69	-0.05
**12**	2.52	11.28	12:16	13:19	01:03	21.98	22.03	0.05

Table S1. Related to Figure 1. The temperatures, timings of the observed OFF and ON state in ATM active mechanics (Fig. 1) and the f_c and amplitude of spontaneous oscillations observed in the ON state. The light phase begins at 11:00 hrs and extends to 23:00 hrs and the dark phase is from 23:00 hrs to 11:00 hrs.

SUPPLEMENTAL EXPERIMENTAL PROCEDURES

ANIMALS AND EXPERIMENTAL SETUP

Oecanthus henryi females were maintained in colonies with *ad libitum* access to food (crushed dog food and pollen) and water under a 12h:12h light:dark cycle. Females were dorsally mounted on a brass bar using a procedure described in Mhatre et al [2, 3]. Experiments were carried out using a previously described setup [2, 3]. The anterior tympanal membrane (ATM) was positioned perpendicular to the laser beam for measurement. As previously reported, only a part of the membrane moves coherently in response to sound (Fig. S1; [2]). The data reported are from this part of the ATM.

All experiments were carried out with ipsilateral sound stimulation with the speaker at 90° to the insect body axis. Acoustic stimuli were produced using the Polytec Scanning Vibrometer software (version 8.8 or 7.4, Polytec Gmbh, Waldbronn, Germany) or synthesised using Matlab (2007b, The Mathworks Inc., Natwick, MA, USA), amplified (Sony Amplifier Model TA- E570; Tokyo, Japan) and passed to a loudspeaker (ESS AMT-1; ESS Laboratory, Inc., Sacramento, CA, USA). Acoustic stimuli were recorded simultaneously and monitored during the experiments using a calibrated 1/8 inch precision pressure microphone (Brüel and Kjær, 4138; Naerum, Denmark) and preamplifier (Brüel and Kjær, 2633). The microphone has a flat response in the measured frequency range and was positioned approximately 5 mm directly above the leg of the animal. Vibration velocities from the tympanal membranes were measured simultaneously using a microscanning laser Doppler vibrometer (Polytec PSV- 400 or PSV-300; Waldbronn, Germany) with an OFV-505 or OFV-056 scanning head fitted with a close-up attachment, and digitised using the Polytec Scanning Vibrometer software (version 8.8 or version 7.4, Polytec Gmbh, Waldbronn, Germany) through a data acquisition board (National Instruments, PCI-6110 or PCI-4452; Austin, TX, USA).

The animal mount, vibrometer and loudspeaker set-up was placed on a vibration isolation table (TMC 784-443-12R; Technical Manufacturing Corp., Peabody, MA, USA). The vibration isolation table and the experimental setup were in an acoustic isolation booth (IAC series 1204A; 4.50 x 2.25 x 1.98 m; Industrial Acoustics, Bronx, NY, USA) separate from the other devices to minimise both acoustic and vibration noise.

EXPERIMENTAL STIMULI: SPONTANEOUS OTOACOUSTIC EMISSIONS

High amplitude spontaneous vibrations of the mechanical system are a well-known feature of active systems. When measuring spontaneous emissions (SOAE), no sound was produced and the behaviour of the ATM and background sound was recorded for at least 12.8 s at 128 kHz. Both the FFT analysis and time traces of the measurement were saved and could be subsequently analysed. If the ATM was in the ON state, the peak frequency of the SOAE was identified from FFT measurements at a resolution of at least 7.8 Hz. The OFF state, when no spontaneous oscillations were observed, allowed us to estimate the passive system noise floor. Both amplitudes and frequencies of spontaneous emissions were different in different individuals indicating the SOAEs were not instrument artefacts. The mean and standard deviation of the frequencies and amplitudes was calculated.

Females were mounted at different times in their light cycle. If no spontaneous oscillations were observed in the frequency spectra, measurements of tympanal frequency response were made immediately at a series of stimulus SPLs. Subsequently, measurements were made at regular intervals and saved when we observed spontaneous oscillations above noise in conspecific frequency range. Details on the timings and temperatures of these measurements are available in Table S1 and Fig. S2. Additionally, the frequencies and amplitudes of the spontaneous oscillations are also reported. Measurements were also made from the ATMs of females injected with DMSO and sacrificed by an abdominal injection of 96% ethanol.

In addition, we also tested the suppression of the SOAEs in response to tones both near and far from SOAE frequency. After SOAEs were recorded on their own, a tone at 4 kHz was played, followed by a tone 100 Hz lower than SOAE frequency. Finally, SOAEs were recorded in silence again (Fig. S2). The female was then administered a glutamate injection (15% by volume with H_2O), both in the abdomen and thorax, in order to eliminate muscles as a potential source of the SOAEs. Following injection of glutamate, a reduction in muscular activity was suggested by tarsal flaccidity, as well as a reduction of breathing movements. SOAEs were recorded both immediately after and 5 minutes after administration of glutamate.

EXPERIMENTAL STIMULI: SPL DEPENDENT FREQUENCY RESPONSE FUNCTION

To test for compressive non-linearity, we measured the frequency response function of the tree cricket ATM. This measurement was done first when no SOAEs were observed in spectra and subsequently again after SOAEs were observed. Periodic chirps were used as acoustic stimuli.

Periodic chirps are analytical signals which require two main parameters to describe them, the frequency band and the frequency resolution. Each periodic chirp consists of a continuous train of tones. The train contains complete cycles of a sine wave at each frequency within the band, spaced at the given resolution. The separation of the frequencies in the stimulus prevents effects from frequency interactions such as two-tone suppression known to occur in active systems [4]. Sampling frequency is determined by the bandwidth being investigated and desired frequency resolution.

Sound pressure level and vibration velocity are used to calculate the amplitude, phase and coherence of the ATM displacement with respect to the sound using standard transfer function formulae [5]. The SPL is kept at the same level across the entire frequency band. For the experiments presented in this manuscript, measurements were made in the frequency band 1.5 to 30 kHz and 1.5 to 10 kHz at a frequency resolution of 62.5 and 12.5 Hz respectively. The sampling rate was 128 kHz and 25.6 kHz respectively. To measure the amplitude dependence of the ATM's frequency response, the sound level was varied from 1 mPa (34 dB SPL) to 40mPa (66 dB SPL) for the wide band signal and 0.25 (21 dB SPL) to 8 mPa (52.04 dB SPL) for the narrower band signal (Fig. S3). The average response to 100 stimulations was calculated for each measurement. To test the amplitude dependence of the ATM in the ON and OFF state, the ATM was tested at 2, 4, 5, 10, 20 and 40 mPa sound pressure levels (n=12 females).

EXPERIMENTAL ANALYSIS: SPL DEPENDENT FREQUENCY RESPONSE FUNCTION

The frequency response of the ATM cannot be completely captured by a single oscillator model [3]. The discovery of a variable low frequency response which has an 'ON/OFF' switch in conjunction with a sensitivity roll off at higher frequencies (Fig. 1D, E) clearly suggests that the system is built from at least two interacting oscillators.

In order to estimate parameters for the two oscillators, we carried out the following fitting exercise on 8 females tested at SPLs of 1, 2, 4, 5, 7, 8, 10, 20 and 40 mPa. At high SPLs, the contribution of the variable oscillator is highly attenuated. This allows us to estimate the displacement amplitude of the ATM due to its passive response, using a forced simple harmonic oscillator (SHO) model.

$$A_\omega = A_0 \frac{{\omega_0}^2}{\sqrt{({\omega_0}^2-\omega^2)^2+(2\gamma\omega_0\omega)^2}} \qquad (1)$$

Where, A_ω is the displacement at angular frequency ω, A_0 is the amplitude of displacement at static force, ω_0 is the angular frequency at resonance, and γ is the damping ratio. The amplitude response was fit to the SHO equation. To estimate the passive resonance, only data above 10 kHz was

included and fits of $R^2 = 0.84 \pm 0.12$ (mean ± SD) were obtained for these 8 females. The passive resonance of the ATM was centred on 16.45± 0.90 kHz and had a damping ratio of 0.23 ± 0.07, or a Q of 2.42 ± 0.85 and finally, A_0 of 5.16 ± 1.64 nm/Pa (all mean ± SD, n=8 females).

Similarly, SHO models were fitted to the amplitude dependent component of ATM sensitivity around the low frequency peak, excluding behaviour above 4 kHz and below 1.75 kHz where coherence is low. Fitting was carried out for responses at 1, 2, 4, 5, 7, 8 and 10 mPa. At higher amplitudes, the low frequency peak was indistinguishable from noise. Data was acquired from the same 8 females and fits had an R^2 of 0.91 ± 0.06 (mean ± SD). The fitted parameters suggested that only the damping ratio changed considerably with stimulus SPL (Fig. S3). Neither f_0 nor $A_{0,}$ both of which are dependent upon stiffness, changed considerably except between 1 and 2mPa. In addition, the two did not covary as expected, suggesting that the observed variation in their values lies within noise (Fig. S3).

EXPERIMENTAL STIMULI: GAIN

We also studied the change in gain using a finer grained time domain approach. The ATM response to an amplitude modulated single frequency tone was measured. The stimulus was 100 ms long with a silence of equivalent length. The amplitude of the main frequency increased linearly over 50 ms, from 0 to a maximum amplitude of 70 mPa at 50 ms, and then decreased linearly to 0 over the following 50 ms. This stimulus allows us to directly measure the change in gain at critical frequency along a continuous range of stimulus amplitude.

The frequency investigated for each specimen was chosen depending on SOAE frequency or the peak frequency of the ATM response, both of which correspond closely to f_c. If SOAEs were observed, the emission frequency was used. If not, the peak frequency observed at 4mPa stimulation was used. A measurement was also made at 7 kHz, a frequency which was always be in the linear regime. Both the acoustic stimulus and the displacement of the ATM were averaged over 500 stimulations, in order to reduce the effect of transient noise present in the measurements. All measurements were made at a sampling frequency of 128 kHz.

EXPERIMENTAL ANALYSIS: GAIN

ATM displacement was calculated from the measured velocity using a built-in function of the Polytec software. Subsequent analysis was performed in Matlab (2010b, The Mathworks Inc., Natwick, MA, USA). The sound pressure was simultaneously measured and recorded. Signals were

filtered only for display, unfiltered and unsmoothed signals were used for fitting. Both signals were filtered using a 3rd order band-pass elliptical filter with a passband peak to peak ripple of 0.5 dB and stopband attenuation of 20 dB. The passband was centred on the stimulus frequency ± 500 Hz. The amplitude envelopes of the signals were calculated using a Hilbert transform [6] and the envelope of the displacement was divided by the sound pressure envelope to calculate instantaneous gain.

The displacement of the ATM near f_c is predicted by the equation describing the gain of a critical oscillator close to a Hopf bifurcation [7]:

$$|x|/|f| \sim |f|^{-2/3} \qquad (2)$$

Where, x is displacement of the oscillator and the f the force applied to it, hence |x|/|f| is the gain of the system. In our case, the force is proportional to the sound pressure level, hence the gain was fitted to the following model:

$$G = C_1 * P^{-2/3} + C_2 \qquad (3)$$

Where, G is the gain, P is the sound pressure envelope, and C_1 and C_2 are constants. C_1 describes the constant relationship between the recorded pressure and the actual force applied to the ATM. C_2 describes the constant gain of the passive linear response of the ATM. During fitting, gain below instantaneous SPL 10 mPa was found to be extremely noisy and was ignored.

To demonstrate the fits across a population (Fig. 2B, Fig. S4), the $P^{-2/3}$component of gain was calculated using the following equation.

$$P_r = (G-C_2) / C_1 \qquad (4)$$

Where, P_r is the said component and all other notation is as in equation 3. Data were downsampled for display, and every 20th point is plotted (Fig. 2B).

EXPERIMENTAL STIMULI: TWO-TONE SUPPRESSION

When excited at f_c in the presence of a second frequency, a CO behaves as if it was not stimulated at f_c and gain is reduced. As a result, sensitivity to a tone can be reduced in the presence of a masking tone, a phenomenon termed two-tone suppression. Additionally, new phantom frequencies, termed distortion products otoacoustic emissions (DPOAEs) are observed in the oscillator behaviour. These behaviours are sensitive to the frequency selectivity of the CO and thus to the relative frequencies and amplitudes of the two tones. The reduction in amplification is usually highest when the two tones are nearly equal in amplitude and similar in frequency [4, 7].

In order to study this non-linear phenomenon, we designed a two-tone stimulus in which the amplitude of the main tone varied continuously with respect to the masking tone. Main tone frequency was determined either by the frequency of the SOAE or the peak of the ATM response at 4 mPa stimulation. All masking tones were 200 Hz above or below the main tone (Fig. S4). The main tone was linearly amplitude modulated like the single frequency ramp. The masking tone was produced at a constant level over the entire 100 ms stimulus duration. The amplitude of the masking tone was a fifth of the maximum amplitude achieved by the main tone (Fig. 3C). Thus, the ratio of the amplitude of the main to masking tone in terms of absolute pressure was varied continuously over 0:1 to 5:1 (Fig. 3C). All measurements were made at a sampling frequency of 128 kHz. After the experiment was conducted with the two-tone stimulus, a measurement was conducted at the same settings and levels with the main tone alone, in order to provide a comparison.

EXPERIMENTAL ANALYSIS: TWO-TONE SUPPRESSION

To measure the frequency content of both the sound and the ATM displacement, power spectral density was calculated at a resolution of 7.8 Hz. The exponent of DPOAE decay [1] was independently estimated for the two sets (Fig. S4). In order to estimate the suppression of the f_c in the presence of a second masking tone, we calculated the gain of main tone in the presence and absence of the masking tone. To isolate the main tone, both the microphone and vibrometer displacement signal were filtered using a 3rd order band pass elliptical filter with a with a passband peak to peak ripple of 0.1 dB and stopband attenuation of 40 dB. The passband was centred on the main tone frequency ± 50 Hz. The same filter centred on the masking frequency was used to estimate the masking tone behaviour. A Hilbert transform allowed the estimation of the envelopes of the filtered signals and the gain for the main frequency was calculated by dividing the displacement amplitude envelope by that of the sound pressure.

EXPERIMENTAL STIMULI: GAIN AND TWO-TONE SUPPRESSION USING CONSTANT AMPLITUDE TONE

Since we observed mild hysteresis in the gain measured using amplitude modulated stimuli, we also measured the ATM displacement using constant amplitude stimuli. The stimuli used were 100 ms long with a constant amplitude tone and a silence of equivalent length. The critical frequency or main frequency was chosen as before either from the frequency of spontaneous emissions or from the ATM response to 4mPa stimulus (Fig. 1D). The ATM was tested at f_c alone at 30 SPLs between 1 and 60 mPa. When testing for two-tone suppression, a second masking tone was produced

simultaneously at a frequency 200 Hz higher than the main frequency at a constant amplitude of 10 mPa.

EXPERIMENTAL ANALYSIS: GAIN AND TWO-TONE SUPPRESSION USING CONSTANT AMPLITUDE TONE

To test for gain, the amplitude of both the ATM displacement and the sound pressure was estimated using a Hilbert transform and then averaged over the entire stimulus length. Gain was calculated and plotted against sound pressure level.

To test for two-tone suppression, the gain from this treatment was compared to gain at f_c in the presence of a masking tone. To isolate the main tone, both the microphone and vibrometer displacement signal were filtered using a 3rd order band pass elliptical filter with a with a passband peak to peak ripple of 0.1 dB and stopband attenuation of 40 dB. The passband was centred on the main tone frequency ± 50 Hz. The amplitude of the filtered main and masking signals was estimated using a Hilbert transform, and averaged across stimulus duration. The power spectral density of the microphone signal and ATM response was calculated for the signal closest to equal amplitude main and masking frequency.

PHYSIOLOGICAL VULNERABILITY: CARBON DIOXIDE INDUCED HYPOXIA

In addition, to investigate the physiological vulnerability of ATM active mechanics, we used carbon dioxide. The effects of CO_2 induced hypoxia are variable and the mechanism of action is unknown. Therefore, we used the two most sensitive tests to address its effects on ATM active mechanics. We studied the gain observed at the critical frequency and the production of distortion products at the same frequency before and after exposure to CO_2 in two separate experiments.

The animal was place in a foam plugged gas chamber into which CO_2 was trickled at a steady rate over 30 minutes. This duration was chosen as it was found that a shorter duration did not reliably produce an effect. In all cases, the animal was anaesthetised after the exposure but revived afterwards as could be observed from a twitch reflex on touching the tarsal segments. Also in all cases, we observed recovery in the active mechanics, albeit at a slower rate than the animal's twitch responses. Only 100 averages were used for this experiment.

SUPPLEMENTAL REFERENCES

1. Barral, J., and Martin, P. (2012). Phantom tones and suppressive masking by active nonlinear oscillation of the hair-cell bundle. Proc. Nat. Acad. Sci. *109*, E1344-E1351.
2. Mhatre, N., Montealegre-Z, F., Balakrishnan, R., and Robert, D. (2009). Mechanical response of the tympanal membranes of the tree cricket *Oecanthus henryi*. J. Comp. Physiol. A *195*, 453-462.
3. Mhatre, N., Bhattacharya, M., Robert, D., and Balakrishnan, R. (2011). Matching sender and receiver: poikilothermy and frequency tuning in a tree cricket. J. Exp. Biol. *214*, 2569-2578.
4. Jülicher, F., Andor, D., and Duke, T. (2001). Physical basis of two-tone interference in hearing. Proc. Nat. Acad. Sci. *98*, 9080-9085.
5. Windmill, J.F.C., Gopfert, M.C., and Robert, D. (2005). Tympanal travelling waves in migratory locusts. J. Exp. Biol. *208*, 157-168.
6. Hartmann, W.H. (1998). Signals, Sound and Sensation, (New York: Springer Verlag).
7. Duke, T.A.J., and Jülicher, F. (2007). Critical oscillators as active elements in hearing: Active processes and otoacoustic emissions in hearing. In Active processes and otoacoustic emissions, Volume 30, G.A. Manley, R.R. Fay and A.N. Popper, eds. (Springer New York), pp. 63-92.
